# Association of average cumulative sensory impairment with falls and hip fractures in older adults: Evidence from a longitudinal study

**DOI:** 10.1016/j.jnha.2026.100895

**Published:** 2026-06-03

**Authors:** Xiongxiong Jing, Shuanglong Hou, Yajing Li

**Affiliations:** a Department of Sport Therapy, Shaanxi Provincial Rehabilitation Hospital, Xi'an, Shaanxi 710065, China; b Department of Rehabilitation Medicine, Tangdu Hospital, Fourth Military Medical University, Xi'an, Shaanxi 710038, China; c Departments of rehabilitation therapy, Division 6, Xi'an TCM Hospital of Encephalopathy, Xi'an, Shaanxi 710032, China

**Keywords:** Auditory impairment, Falls, Hip fractures, Sensory impairment, Visual impairment

## Abstract

**Background:**

Sensory impairment is prevalent among older adults and a recognized risk factor for falls and fractures. However, prior studies treated sensory impairment as a static, point-in-time exposure, overlooking its progressive nature. This study investigated the association between the long-term, cumulative burden of sensory impairment and risks of falls and hip fractures in older adults.

**Methods:**

This longitudinal investigation included 3391 participants aged ≥60 years from the China Health and Retirement Longitudinal Study (CHARLS). Average cumulative sensory impairment scores (dual, visual, and auditory) were calculated across four waves (2011–2018) using the area under the curve. Incident falls and hip fractures were assessed through self-report during follow-up (2013–2020). Cox proportional hazards models and restricted cubic spline analyses evaluated associations.

**Results:**

During follow-up, 1576 falls and 189 hip fractures occurred. In fully adjusted models, compared with the lowest quartile (Q1), participants in the highest quartile (Q4) of average cumulative dual sensory impairment exhibited a 36% higher fall risk (HR = 1.36, 95% CI: 1.18–1.58) and a 116% higher hip fracture risk (HR = 2.16, 95% CI: 1.36–3.45). Significant linear trends were evident for both outcomes (P for trend <0.01). Similar graded associations were found for visual and auditory impairment separately. Restricted cubic spline analyses confirmed monotonic, linear dose-response relationships.

**Conclusion:**

The long-term burden of sensory impairment is independently and dose-dependently associated with increased risks of falls and hip fractures. These findings highlight the importance of dynamic sensory monitoring and integrating sensory health management into prevention strategies.

**Clinical trial number:**

not applicable.

## Highlights

• Average cumulative sensory impairment demonstrates a dose-dependent association with increased fall and hip fracture risks.
• The highest quartile of dual sensory impairment raised fall risk by 36% and hip fracture risk by 116%.
• Findings advocate for dynamic, long-term dual sensory monitoring in fall and hip fracture prevention.

## 1 Introduction

The global aging population is developing at an unprecedented pace, and the attendant age-related diseases burden has emerged as​ a key public health challenge in the 21st century [1]. Within the scope of geriatric syndrome, falls and their devastating sequelae, particularly hip fractures, pose a serious threat to​ the quality of life of older adults [2]. Hip fracture is often termed the “last fracture in life,” characterized by high mortality, difficulty in recovering function, relatively high medical expenses [[3], [4], [5]]. Epidemiological prediction underscores the scale of hip fractures. In 2011, the age-standardized incidence rate among individuals aged ≥ 55 years reached 948.81 per 100,000, with global cases projected to exceed 6.3 million by 2050 [6]. Crucially, about 88% of hip fractures are directly attributable to falls [7]. Therefore, identifying modifiable risk factors for falls and fractures is crucial for effectively preventing and reducing the associated societal burden.

Sensory impairment, particularly in vision and hearing, is highly prevalent among older adults, with incidence rates increasing markedly with advancing age [8]. Data from China show that over half of the older adults experience visual or auditory impairments, with 45.4% having dual sensory impairment [9]. A growing body of evidence consistently links single and dual sensory impairments to an increased risk of falls and related injuries [[9], [10], [11]]. The underlying mechanisms may involve deficits in postural control, decreased balance function, and impaired cognitive resources [9,11].

Despite existing evidence, there are clear methodological limitations: most studies have relied on single or limited baseline assessments, treating sensory impairment as a static, point-in-time exposure. However, the decline in visual and auditory functions is a progressive process characterized by gradual accumulation​ throughout the life course. This long-term cumulative burden may exert a more profound impact on physiological reserves and functional abilities than a single baseline impairment. While recent studies have examined the detrimental effects of average cumulative sensory impairment on cognitive and mental health [12], its specific association with the risk of falls and hip fractures remains largely uninvestigated.

To address this gap, this study quantifies the long-term burden of sensory decline using average cumulative impairment as the exposure metric. We aim to investigate the dose-response relationships between the cumulative visual, auditory, and dual sensory impairment and the subsequent risks of falls and fractures. The findings are expected to provide novel evidence for developing targeted strategies to prevent fall and hip fracture in older adults.

## 2 Methods

### 2.1 Study design and participants

This study utilized data from the China Health and Retirement Longitudinal Study (CHARLS), a nationally representative longitudinal survey aimed at assessing the health status and socioeconomic characteristics of middle-aged and older adults aged 45 years and above in China. The baseline survey was conducted in 2011, followed by a follow-up evaluation every two years thereafter. To date, five waves of data have been publicly released.

Using 2011 as the baseline, an initial sample of 17,708 participants was enrolled. Since sensory impairment data were not collected in the 2020 wave, the exposure was defined based on data from four survey waves (2011–2018), and outcome follow-up was extended through 2020. The sample selection criteria included: (1) age ≥60 years at baseline; (2) completion of sensory function assessments at baseline and during subsequent follow-ups; and (3) no self-reported history of falls or hip fracture at baseline. Additionally, participants who were lost to follow-up or died during the follow-up period were excluded. **Fig.** S1 illustrates the selection process of participants. The study protocol was approved by the Biomedical Ethics Review Committee of Peking University (IRB00001052-11015), and written informed consent was obtained from all participants.

### 2.2 Assessment of average cumulative sensory impairment

Sensory function was evaluated using self-reported information. Auditory function was evaluated via the question: “Would you say your hearing is excellent, very good, good, fair, or poor?”. Visual function was assessed through two questions: “How good is your eyesight for seeing things at a distance?” and “How good is your eyesight for seeing things up close?”. Each question has five response options: (1) excellent, (2) very good, (3) good, (4) fair, and (5) poor, assigned 1–5 points respectively. By adding visual and auditory scores, a dual sensory impairment score ranging from 2 to 10 is obtained, with higher scores indicating poorer sensory function.

To quantify long-term cumulative exposure to sensory impairment, we calculated the average cumulative sensory impairment score based on data from the four survey waves in 2011, 2013, 2015, and 2018. This approach, applying the concept of area under the curve (AUC), provides a more robust reflection of sustained exposure over time [12,13]. The average cumulative sensory impairment score was specifically calculated using the following formula:

$$\text{A}\text{v}\text{e}\text{r}\text{a}\text{g}\text{e}\text{C}\text{u}\text{m}\text{u}\text{l}\text{a}\text{t}\text{i}\text{v}\text{e}\text{S}\text{e}\text{n}\text{s}\text{o}\text{r}\text{y}\text{I}\text{m}\text{p}\text{a}\text{i}\text{r}\text{m}\text{e}\text{n}\text{t}=\frac{\left(\frac{\left(\text{Y}1+\text{Y}2\right)\times 2}{2}+\frac{\left(\text{Y}2+\text{Y}3\right)\times 2}{2}+\frac{\left(\text{Y}3+\text{Y}4\right)\times 3}{2}\right)}{7}$$

Where Y_1_, Y_2_, Y_3_, and Y_4_ represent the sensory impairment scores for 2011, 2013, 2015, and 2018, respectively (**Fig.** S2). The trapezoidal area for each consecutive interval was computed and summed to obtain the total AUC, which was then divided by the total follow-up duration of 7 years to derive the average cumulative sensory impairment score.

### 2.3 Assessment of falls and hip fractures

The primary outcome variables in this study were incident falls and hip fractures occurring between 2013 and 2020. Participants were asked the following two questions: “Have you fallen down since the last follow-up?” and “Have you fractured your hip since the last follow-up?”. An affirmative response to these two questions was recorded as a positive event.

### 2.4 Covariates

Potential confounding covariates were selected based on the existing studies and assessed at the baseline survey [[14], [15], [16]]. These covariates included sociodemographic factors, health-related lifestyle, chronic disease conditions, and hearing aid use [17]. Sociodemographic factors included age, sex (male/female), residence (urban/rural), marital status (married/others), and educational attainment (illiterate/junior school and below/high school and above). Health-related lifestyle factors included smoking status (never/ever), drinking status (never/ever), and social participation (yes/no). Social participation was defined as engagement in any of the 11 social activities listed in the CHARLS questionnaire. Chronic diseases were identified based on self-reported physician diagnoses and included hypertension, heart disease, stroke, chronic lung disease, arthritis or rheumatism, kidney disease, digestive system diseases, diabetes, and memory-related diseases. Additionally, medication use for hypertension, diabetes, and dyslipidemia were also considered as potential covariates [18,19].

### 2.5 Statistical analysis

Baseline characteristics of the study participants are presented as means ± standard deviations (SDs) for continuous variables and frequencies (percentages) for categorical variables. Missing covariate data, as detailed in **Table** S1, were assumed to be missing at random and handled using multiple imputation with chained equations (MICE). Differences in baseline characteristics across quartiles of average cumulative dual sensory impairment were compared using one-way analysis of variance (ANOVA) for continuous variables and the chi-square test for categorical variables.

Cox proportional hazards regression models were employed to evaluate the associations between average cumulative sensory impairment (dual, visual, and auditory) and incident falls and hip fractures, and hazard ratios (HRs) with 95% confidence intervals (CIs) were estimated. Sensory impairment was analyzed both as a categorical variable (divided into quartiles, Q1–Q4) and a continuous measure (per-quartile and per-SD increment). Four models were constructed: Model 1 was the crude, unadjusted analysis; Model 2 incorporated adjustments for sociodemographic factors (age, sex, residence, marital status, and educational attainment); Model 3 further included health-related lifestyle variables; Model 4 additionally adjusted for all chronic conditions. Given the negligible prevalence of hearing aid use (0.62%) leading to zero events in certain subgroups, this variable was excluded from the final models to prevent complete separation. The proportional hazards assumption was examined via Schoenfeld residuals. Tests for linear trend across quartiles were conducted by modeling the quartile variable as a continuous term.

Kaplan-Meier survival curves were generated to visualize the cumulative incidence of falls and hip fractures across quartiles of average cumulative sensory impairment, and the log-rank test was employed to compare survival distributions. Restricted cubic spline analysis was used to model the dose-response relationship between continuous sensory impairment scores and the hazard of the outcomes, testing for potential non-linearity.

To evaluate the consistency of the findings, subgroup analyses were conducted according to age (<65 vs. ≥65 years), sex, residence, marital status, educational attainment, smoking and drinking status, social participation, and chronic disease burden (0–1 vs. ≥2 diseases). Interaction terms were included to test for effect modification. Sensitivity analyses were conducted to assess robustness: (1) replicating the main analysis in a complete‑case dataset; (2) calculating E‑values​ to quantify the potential impact of unmeasured confounding; and (3) additionally adjusting​ for medication use related to hypertension, diabetes, and dyslipidemia in the fully adjusted model.

All analyses were implemented in R 4.3.0 and SPSS 27.0. Statistical significance was defined as a two‑sided P < 0.05.

## 3 Results

### 3.1 Baseline characteristics of participants

A total of 3391 participants were enrolled in the final analysis. Their baseline characteristics, categorized by quartiles of average cumulative dual sensory impairment are presented in Table 1. The cohort had an average age of 66.57 ± 5.62 years; 51.1% were male and 64.6% lived in rural areas. Relative to those in the lowest quartile, participants in the highest quartile were older, included a greater proportion of females, were more frequently rural residents, attained lower education levels, and engaged less in social activities. They also showed a significantly higher burden of chronic comorbidities, such as hypertension, heart disease, chronic lung disease, arthritis or rheumatism, kidney disease, and digestive diseases (all P < 0.05).

**Table 1:** Baseline characteristics of participants stratified by average cumulative dual sensory impairment.

Characteristics	Overall (n = 3391)	Average cumulative dual sensory impairment				P-value
		Q1 (n = 846)	Q2 (n = 808)	Q3 (n = 886)	Q4 (n = 851)	
Age, years	66.57 ± 5.62	66.17 ± 5.59	66.47 ± 5.58	66.26 ± 5.41	67.39 ± 5.83	< 0.001
Sex						< 0.001
Male	1732 (51.1)	469 (55.4)	413 (51.1)	467 (52.7)	383 (45.0)	
Female	1659 (48.9)	377 (44.6)	395 (48.9)	419 (47.3)	468 (55.0)	
Residence						< 0.001
Rural	2191 (64.6)	498 (58.9)	506 (62.6)	580 (65.5)	607 (71.3)	
Urban	1200 (35.4)	348 (41.1)	302 (37.4)	306 (34.5)	244 (28.7)	
Marital status						0.175
Married	2797 (82.5)	701 (82.9)	676 (83.7)	739 (83.4)	681 (80.0)	
Others	594 (17.5)	145 (17.1)	132 (16.3)	147 (16.6)	170 (20.0)	
Education						< 0.001
Illiterate	1830 (54.0)	418 (49.4)	413 (51.1)	471 (53.2)	528 (62.0)	
Junior school and below	895 (26.4)	185 (23.0)	232 (28.7)	258 (29.1)	210 (24.7)	
High school and above	666 (19.6)	233 (27.5)	163 (20.2)	157 (17.7)	113 (13.3)	
Smoking status						0.085
Never	1955 (57.7)	474 (56.0)	483 (59.8)	488 (55.1)	510 (59.9)	
Ever	1436 (42.3)	372 (44.0)	325 (40.2)	398 (44.9)	341 (40.1)	
Drinking status						0.010
Never	2037 (60.1)	491 (58.0)	687 (60.3)	509 (57.4)	550 (64.6)	
Ever	1354 (39.9)	355 (42.0)	321 (39.7)	377 (42.6)	301 (35.4)	
Social participation						< 0.001
Yes	1646 (48.5)	443 (52.4)	411 (50.9)	428 (48.3)	364 (42.8)	
No	1745 (51.5)	403 (47.6)	397 (49.1)	458 (51.7)	487 (57.2)	
Hypertension	1049 (30.9)	225 (26.6)	231 (28.6)	296 (33.4)	297 (34.9)	< 0.001
Heart disease	483 (14.2)	90 (10.6)	101 (12.5)	141 (15.9)	151 (17.7)	< 0.001
Stroke	94 (2.8)	16 (1.9)	21 (2.6)	27 (3.0)	30 (3.5)	0.207
Chronic lung disease	383 (11.3)	73 (8.6)	81 (10.0)	103 (11.6)	126 (14.8)	< 0.001
Arthritis or rheumatism	1213 (35.8)	206 (24.3)	270 (33.4)	342 (38.6)	395 (46.4)	< 0.001
Kidney disease	180 (5.3)	26 (3.1)	38 (4.7)	53 (6.0)	63 (7.4)	0.001
Digestive disease	744 (21.9)	122 (14.4)	167 (20.7)	214 (24.2)	241 (28.3)	< 0.001
Diabetes	210 (6.2)	42 (5.0)	41 (5.1)	64 (7.2)	63 (7.4)	0.053
Memory-related disease	58 (1.7)	8 (0.9)	15 (1.9)	14 (1.6)	21 (2.5)	0.110
Medication for hypertension	761 (22.64)	167 (19.74)	163 (20.17)	220 (24.83)	211 (2.48)	0.009
Medication for diabetes	108 (3.18)	20 (2.36)	23 (2.85)	32 (3.61)	33 (3.88)	0.197
Medication for dyslipidemia	158 (4.66)	29 (3.43)	37 (4.58)	45 (5.08)	47 (5.52)	0.262
Hearing aids use	21 (0.62)	3 (0.35)	5 (0.62)	4 (0.45)	9 (1.06)	0.259

Q1, quartile 1; Q2, quartile 2; Q3, quartile 3; Q4, quartile 4.

### 3.2 Association between average cumulative sensory impairment and risk of falls

**Fig. 1:**
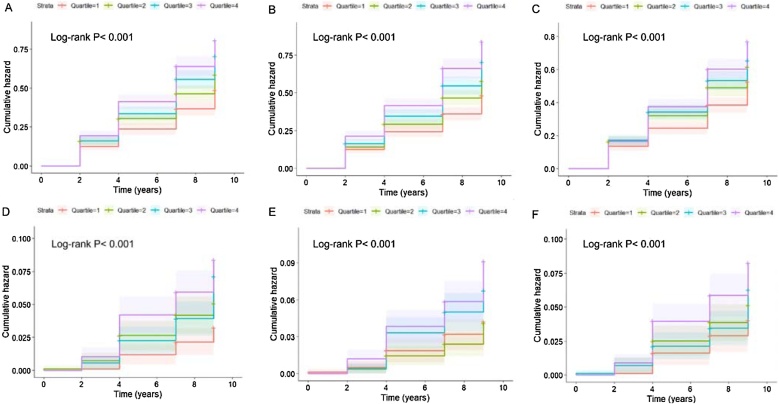
Kaplan–Meier curves for cumulative risks of falls and hip fractures by average cumulative sensory impairment. A, Average cumulative dual sensory impairment and falls risk; B, Average cumulative visual sensory impairment and falls risk; C, Average cumulative auditory sensory impairment and falls risk; D, Average cumulative dual sensory impairment and hip fractures risk; E, Average cumulative visual sensory impairment and hip fractures risk; F, Average cumulative auditory sensory impairment and hip fractures risk.

During the follow-up period, a total of 1576 fall incidents were recorded. Kaplan-Meier survival curves demonstrated a significant elevation in cumulative fall risk with increasing quartiles of average cumulative dual sensory impairment (log-rank test, P < 0.001; Fig. 1A). As shown in Table 2, the fully adjusted model revealed a significant positive dose-response relationship between the severity of dual sensory and fall risk: compared with participants in the lowest quartile (Q1), those in the highest quartile (Q4) had a 36% increased risk of falls (HR = 1.36, 95% CI: 1.18–1.58). A linear trend test indicated a 12% increase in fall risk per quartile increment (per-quartile HR = 1.12, 95% CI: 1.07–1.18); for each 1-SD increase in average cumulative dual sensory impairment, the fall risk increased by 15% (per-SD HR = 1.15, 95% CI: 1.09–1.21).

**Table 2:** Association between average cumulative sensory impairment and risk of falls.

Sensory impairment	Falls, Cases/Events	HR, 95% CI			
		Model 1	Model 2	Model 3	Model 4
Dual sensory impairment					
Quartile 1	319/846	Reference	Reference	Reference	Reference
Quartile 2	351/808	1.21 (1.04, 1.40) *	1.18 (1.01, 1.37) *	1.18 (1.01, 1.37) *	1.12 (0.96, 1.31)
Quartile 3	441/886	1.40 (1.22, 1.63) ***	1.39 (1.20, 1.61) ***	1.38 (1.20, 1.60) ***	1.29 (1.12, 1.50) **
Quartile 4	465/851	1.61 (1.40, 1.86) ***	1.52 (1.31, 1.75) ***	1.52 (1.31, 1.75) ***	1.36 (1.18, 1.58) ***
Test for linear trend	–	1.17 (1.12, 1.22) ***	1.15 (1.10, 1.20) ***	1.16 (1.11, 1.22) ***	1.12 (1.07, 1.18) ***
Per SD increment	–	1.19 (1.14, 1.25) ***	1.17 (1.11, 1.23) ***	1.19 (1.13, 1.26) ***	1.15 (1.09, 1.21) ***
Visual sensory impairment					
Quartile 1	298/799	Reference	Reference	Reference	Reference
Quartile 2	416/964	1.20 (1.03, 1.39) *	1.20 (1.04, 1.40) *	1.19 (1.03, 1.39) *	1.15 (0.99, 1.34)
Quartile 3	400/802	1.42 (1.22, 1.65) ***	1.40 (1.20, 1.63) ***	1.40 (1.20, 1.62) ***	1.29 (1.11, 1.50) **
Quartile 4	462/864	1.68 (1.45, 1.92) ***	1.58 (1.36, 1.83) ***	1.57 (1.35, 1.82) ***	1.43 (1.23, 1.66) ***
Test for linear trend	–	1.19 (1.14, 1.24) ***	1.16 (1.11, 1.22) ***	1.16 (1.11, 1.21) ***	1.13 (1.08, 1.18) ***
Per SD increment	–	1.23 (1.16, 1.29) ***	1.19 (1.13, 1.26) ***	1.19 (1.13, 1.26) ***	1.15 (1.09, 1.21) ***
Auditory sensory impairment					
Quartile 1	324/810	Reference	Reference	Reference	Reference
Quartile 2	380/844	1.18 (1.02, 1.37) *	1.15 (1.00, 1.34)	1.15 (0.99, 1.33)	1.12 (0.96, 1.30)
Quartile 3	408/859	1.26 (1.09, 1.45) **	1.25 (1.08, 1.45) **	1.24 (1.08, 1.44) **	1.18 (1.02, 1.39) *
Quartile 4	464/878	1.43 (1.24, 1.64) ***	1.37 (1.19, 1.58) ***	1.37 (1.19, 1.58) ***	1.26 (1.09, 1.46) **
Test for linear trend	–	1.12 (1.07, 1.17) ***	1.11 (1.06, 1.16) ***	1.11 (1.06, 1.16) ***	1.08 (1.03, 1.13) **
Per SD increment	–	1.13 (1.08, 1.19) ***	1.12 (1.07, 1.18) ***	1.12 (1.06, 1.18) ***	1.09 (1.03, 1.14) **

Model 1, unadjusted; Model 2, adjusted for age, sex, residence, marital status, and education; Model 3, further adjusted for smoking status, drinking status, and social participation; Model 4, additionally adjusted for hypertension, heart disease, stroke, chronic lung disease, arthritis or rheumatism, kidney disease, digestive disease, diabetes, and memory-related disease. *P < 0.05; **P < 0.01; ***P < 0.001.

Average cumulative visual and auditory sensory impairments each exhibited similar graded associations with fall risk. For visual impairment, the HR for Q4 versus Q1 was 1.43 (95% CI: 1.23–1.66), with a per-quartile trend HR of 1.13 (95% CI: 1.08–1.18); cumulative risk curves differed significantly across groups (log-rank test, P < 0.001; Fig. 1B). For auditory impairment, the HR for the highest quartile relative to the lowest quartile was 1.26 (95% CI: 1.09–1.46), with a per-quartile trend HR of 1.08 (95% CI: 1.03–1.13); similarly, cumulative risk curves differed significantly among auditory impairment subgroups (log-rank test, P < 0.001; Fig. 1C).

### 3.3 Association between average cumulative sensory impairment and risk of hip fractures

A total of 189 incident hip fracture cases were identified. Kaplan-Meier cumulative risk curves indicated that the cumulative risk of hip fracture was progressively elevated with increasing quartiles of average cumulative sensory impairment (log-rank test, P < 0.001; Fig. 1D). In the fully adjusted model (Table 3), participants with dual sensory impairment in Q4 had a 2.16-fold higher risk of hip fracture compared to those in Q1 (HR = 2.16, 95% CI: 1.36–3.45). A significant linear trend was observed across quartiles (per-quartile HR = 1.27, 95% CI: 1.11–1.45), and each SD increase in dual sensory impairment was associated with a 43% increased risk of hip fracture (HR = 1.43, 95% CI: 1.21–1.69).

**Table 3:** Association between average cumulative sensory impairment and risk of hip fractures.

Sensory impairment	Hip fractures, cases/events	HR, 95% CI			
		Model 1	Model 2	Model 3	Model 4
Dual sensory impairment					
Quartile 1	26/846	Reference	Reference	Reference	Reference
Quartile 2	39/808	1.60 (0.98, 2.63)	1.59 (0.97, 2.62)	1.60 (0.97, 2.64)	1.56 (0.94, 2.56)
Quartile 3	58/886	2.15 (1.36, 3.42) **	2.09 (1.32, 3.33) **	2.08 (1.31, 3.30) **	2.02 (1.26, 3.23) **
Quartile 4	66/851	2.59 (1.65, 4.08) **	2.25 (1.42, 3.55) **	2.29 (1.45, 3.61) ***	2.16 (1.36, 3.45) **
Test for linear trend	–	1.35 (1.18, 1.54) ***	1.35 (1.18, 1.54) ***	1.28 (1.13, 1.47) **	1.27 (1.11, 1.35) **
Per SD increment	–	1.54 (1.31, 1.82) ***	1.45 (1.23, 1.70) ***	1.45 (1.23, 1.71) ***	1.43 (1.21, 1.69) ***
Visual sensory impairment					
Quartile 1	32/799	Reference	Reference	Reference	Reference
Quartile 2	37/964	0.95 (0.59, 1.53)	0.99 (0.61, 1.58)	0.97 (0.60, 1.56)	0.96 (0.60, 1.55)
Quartile 3	51/802	1.58 (1.02, 2.47) *	1.57 (1.01, 2.44) *	1.58 (1.01, 2.45) *	1.49 (0.95, 2.34)
Quartile 4	69/864	2.12 (1.39, 3.22) ***	1.83 (1.20, 2.79) **	1.83 (1.20, 2.79) **	1.74 (1.13, 2.67) *
Test for linear trend	–	1.34 (1.17, 1.53) **	1.26 (1.11, 1.44) **	1.27 (1.11, 1.45) **	1.24 (1.09, 1.43) **
Per SD increment	–	1.45 (1.23, 1.71) ***	1.35 (1.15, 1.58) **	1.39 (1.15, 1.59) ***	1.32 (1.12, 1.55) **
Auditory sensory impairment					
Quartile 1	31/810	Reference	Reference	Reference	Reference
Quartile 2	41/844	1.29 (0.81, 2.04)	1.26 (0.79, 2.01)	1.27 (0.79, 2.02)	1.24 (0.77, 1.98)
Quartile 3	50/859	1.53 (0.98, 2.39)	1.55 (0.99, 2.43)	1.56 (1.00, 2.45)	1.53 (0.97, 2.40)
Quartile 4	67/878	2.05 (1.34, 3.14) **	1.83 (1.19, 2.81) **	1.85 (1.21, 2.83) **	1.77 (1.15, 2.73) *
Test for linear trend	–	1.26 (1.11, 1.44) ***	1.22 (1.07, 1.39) **	1.22 (1.07, 1.39) **	1.21 (1.06, 1.38) **
Per SD increment	–	1.42 (1.21, 1.66) ***	1.36 (1.13, 1.59) ***	1.37 (1.17, 1.60) ***	1.35 (1.15, 1.58) **

Model 1, unadjusted; Model 2, adjusted for age, sex, residence, marital status, and education; Model 3, further adjusted for smoking status, drinking status, and social participation; Model 4, additionally adjusted for hypertension, heart disease, stroke, chronic lung disease, arthritis or rheumatism, kidney disease, digestive disease, diabetes, and memory-related disease. *P < 0.05; **P < 0.01; ***P < 0.001.

For average cumulative visual impairment, the risk of hip fracture was significantly elevated in Q4 (HR = 1.74, 95% CI: 1.13–2.67), with evidence of a positive linear trend (per-quartile HR = 1.24, 95% CI: 1.09–1.43). Cumulative hip fracture risk differed significantly among the quartile groups (log-rank test, test, p < 0.001; Fig. 1E). Regarding average cumulative auditory impairment, the risk of hip fracture was also significantly higher in the highest quartile (HR = 1.77, 95% CI: 1.15–2.73), and a linear trend was observed (per-quartile HR = 1.21, 95% CI: 1.06–1.38). The log-rank test, test for cumulative risk curves was statistically significant (P < 0.001, Fig. 1F), indicating an ascending risk with increasing quartiles.

### 3.4 Restricted cubic spline analysis

**Fig. 2:**
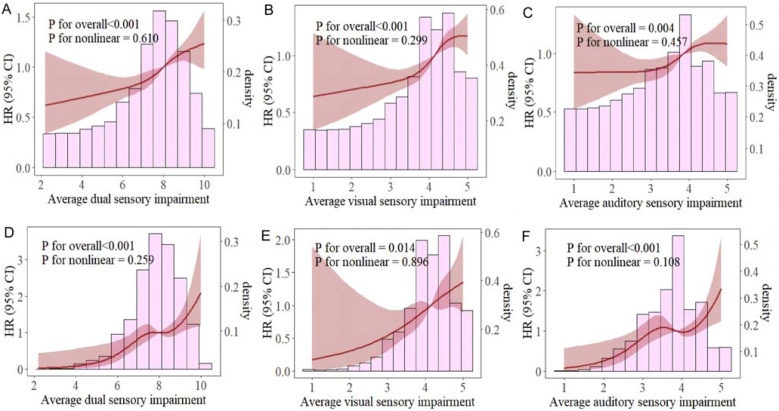
Restricted cubic spline curve of average cumulative sensory impairment with risk of falls and hip fractures. All models were adjusted for age, sex, residence, marital status, education, smoking status, drinking status, social participation, hypertension, heart disease, stroke, chronic lung disease, arthritis or rheumatism, kidney disease, digestive disease, diabetes, and memory-related disease. A, Average cumulative dual sensory impairment and falls risk; B, Average cumulative visual sensory impairment and falls risk; C, Average cumulative auditory sensory impairment and falls risk; D, Average cumulative dual sensory impairment and hip fractures risk; E, Average cumulative visual sensory impairment and hip fractures risk; F, Average cumulative auditory sensory impairment and hip fractures risk.

To explore potential nonlinear dose-response relationships between levels of average cumulative sensory impairment and the risks of the outcomes, we performed restricted cubic spline analyses. As shown in Fig. 2, monotonic linear dose-response relationships were observed between all three types of average cumulative sensory impairment (dual, visual, and auditory) and the risks of both falls and hip fractures. The P-values for nonlinearity across all spline curves were greater than 0.05, indicating the absence of apparent threshold effects or nonlinear associations.

### 3.5 Subgroup analysis and sensitivity analysis

Extensive subgroup analyses were performed to evaluate the consistency of associations between average cumulative sensory impairment and the risks of falls and hip fractures across different population subgroups (**Table** S1–S2). The results showed that the positive associations of average cumulative sensory impairment (dual, visual, and auditory) with the risks of both outcomes were generally consistent across all subgroups. Tests for interaction were not statistically significant in any subgroup (all P for interaction > 0.05), indicating that the observed associations were robust across diverse population strata.

Three sensitivity analyses were performed. First, the primary analyses were repeated using a complete-case dataset, and the results were highly consistent in both direction and magnitude with those obtained from the imputed sample (**Table** S3–S4). Second, E-values were calculated for the observed associations (**Table** S5–S6). The relatively large E-values suggest that an unmeasured confounder of considerable strength, simultaneously associated with both the exposure and the outcome, would be required to fully explain away the observed associations. Third, upon further adjusting for medication use in Model 4, the results remained robust, with only minimal changes in HRs (e.g., HR for dual sensory impairment, the HR for Q4 vs. Q1 changed from 1.36 to 1.35 for falls, and from 2.16 to 2.18 for hip fractures; **Table** S7).

## 4 Discussion

This large-scale longitudinal study investigated the association between the long-term burden of sensory impairments and the risk of falls and hip fractures. Our findings indicate that a higher average cumulative burden of sensory impairments is significantly associated with increased risks of falls and hip fractures, demonstrating a significant linear dose-response relationship. This association was consistently observed for visual, auditory, and dual sensory impairment, with the strongest association observed for dual sensory impairment. By introducing the concept of average cumulative exposure, this study enhances the understanding of how long-term sensory decline contributes to falls and hip fractures in the older population.

This study extends the existing research linking sensory impairment to higher risk of falls and fractures. Numerous prospective studies have consistently identified sensory impairment as a significant risk factor for falls and hip fractures. For instance, a recent study of U.S. Medicare beneficiaries found that individuals with both vision and hearing problems had a 45% higher rates of falls, along with increased fear of falling [10]. Another long-term survey in South Korea reported that severe hearing loss increases hip fractures risk by 67% [20]. Among patients aged 65 or older with hip fracture, visual impairment affected 15.4%, hearing impairment 38.6%, and dual impairments 30.1% [21]. While our study aligns with these findings, it offers a crucial methodological advancement. Prior research often treated sensory impairment as a static characteristic measured at a single time point. However, sensory function declines gradually and nonlinearly with age [22]. The concept of average cumulative sensory impairment captures this progressive decline, representing the sustained physiological load or sensory burden over time [12,13]. Compared to a cross-sectional assessment, this measure may better reflect long-term depletion of physiological reserve, thereby potentially confer greater predictive power for long-term adverse outcomes.

Several plausible mechanisms may explain the association between cumulative sensory impairment and the risks of falls and fractures. Primarily, sensory impairment directly compromises sensory input and diminishes central integration efficiency [[23], [24], [25]]. Hearing loss not only impairs auditory and spatial awareness of the immediate surroundings but also imposes an increased cognitive load for auditory processing [23,26], which competitively diverts attentional resources essential for maintaining postural stability [27]. Concurrently, visual impairment restricts visual feedback, leading to impaired postural control, gait abnormalities, and reduced contrast sensitivity [28]. The compounding of these deficits can severely undermine multisensory integration, resulting in increased gait variability [29] and diminished balance control [30], thereby constituting a direct biomechanical risk for falls and fractures. Furthermore, communication difficulties and reduced social engagement triggered by sensory impairment often lead to a significant decline in physical activity [31]. This, in turn, contributes to disuse-related physiological deterioration, such as sarcopenia [32] and osteoporosis [33], fundamentally weakening the functional reserve of the musculoskeletal system and its capacity to resist falls and mitigate fracture risk. Moreover, sensory impairment can foster a strong fear of falling, a well-established and potent risk factor for falls [10,34]. This fear often prompts individuals to adopt maladaptive behavioral strategies, such as trigger activity restriction and social withdrawal, resulting in deconditioning, muscle weakness, and further deterioration of balance and gait, thereby establishing a vicious cycle that heightens the risk of falls.

As the global population ages and age-related sensory impairment becomes increasingly common, preventing falls and fractures in older adults is an urgent public health priority. Our findings suggest that clinical and public health interventions should move beyond assessing clinically defined sensory impairment at isolated time points. Instead, a systematic approach for the long-term, dynamic monitoring of sensory function in older adults should be implemented. Individuals exhibiting progressive cumulative sensory decline, even before reaching the threshold of a clinical diagnosis, should be identified as high risk for falls and integrated into early targeted intervention programs. Evidence-based interventions should be multifaceted. First, optimizing sensory input is essential, including the timely provision and proper fitting of visual and auditory aids, combined with structured sensory training to maximize remaining function [17,35]. Second, environmental modifications can reduce risk, home safety assessments should be used to improve lighting, reduce glare, remove tripping hazards, and enhance the acoustic environment [36]. In addition, tailored exercise programs that improve lower-limb strength, balance, gait stability, and proprioception have proven effective for fall prevention [37]. For individuals with dual sensory impairment, there is a pressing need to develop and validate targeted, multidimensional, and intensive intervention protocols.

This study has several strengths, including its prospective, nationally representative cohort design, which enhances robustness in examining causal relationships. The innovative application of an average cumulative exposure model facilitates more precise quantification of the long-term burden associated with sensory decline. However, several limitations must be acknowledged. First, sensory function was self-reported, which may introduce measurement error and recall bias. In particular, existing evidence suggests that self-reported visual difficulty has limited validity in predicting objectively measured visual impairment among older adults [38]. Future studies incorporating objective measurements would enable more accurate exposure classification. Second, the negligible prevalence of hearing aid use in our cohort (0.62%) precluded meaningful analysis​ of its potential effect modification on fall or fracture risk. This likely underestimates the protective effect​ of sensory interventions (e.g., hearing rehabilitation), as hearing aid use could theoretically reduce fall risk by improving environmental awareness [17]. Future investigations in populations with higher hearing aid coverage​ are warranted to explore this association. Third, although the calculated E-values suggest that the observed associations are relatively robust to unmeasured confounding, residual confounding may persist. Factors such as detailed medication history (e.g., sedatives and sleeping pills) [39], vestibular function [40], and specific home environmental risks [41] were not fully captured. While sensitivity analyses adjusting for select fall-related medications yielded consistent results, the absence of granular data on dosage and duration limits our ability to fully account for pharmacological effects. Future research should collect detailed prescription records to enable more rigorous control of medication-related confounding. Furthermore, outcome events were also primarily self-report, which may result in underreporting, especially for minor falls that did not lead to medical attention. Such nondifferential misclassification, however, would more likely attenuate rather than exaggerate the true effect estimates [42]. Finally, as the study population were older adults in China, caution is warranted when generalizing the conclusions to populations with different racial, ethnic, or cultural backgrounds.

## 5 Conclusion

In conclusion, this longitudinal study demonstrates that the increasing burden of average cumulative sensory impairment significantly and independently elevates the risk of falls and hip fractures among older adults in China. These findings suggest that sensory health should be conceptualized as a dynamic continuum, and monitoring its cumulative decline over the life course is of considerable importance. Integrating routine sensory assessment and management into clinical and public health practice holds significant potential for reducing the personal and societal burdens associated with falls and hip fractures.

## Authors' contributions

X.-X.J. was involved in conceptualization, formal analysis, investigation, visualization, writing—original draft, and writing—review and editing. S.-L.H. was involved in formal analysis, writing—original draft, and writing—review and editing. Y.-J.L. contributed to conceptualization, funding acquisition, supervision, and writing—review and editing.

## Ethics approval and consent to participate

The entire study process adhered to the Declaration of Helsinki, and the study results were reported following the STROBE guidelines. The protocol for the CHARLS cohort was authorized by the Ethics Review Committee of Peking University (IRB00001052–11015), and all participants provided written informed consent at the time of participation.

## Consent for publication

Not applicable.

## Declaration of Generative AI and AI-assisted technologies in the writing process

I have not used any AI at all.

## Funding

This research did not receive any specific grant from funding agencies in the public, commercial, or not-for-profit sectors.

## Data availability

CHARLS datasets are available for download at the CHARLS home website: http://charls.pku.edu.cn.

## Declaration of competing interest

I have nothing to declare.
