# Supplementary material for: Association of average cumulative sensory impairment with falls and hip fractures in older adults: Evidence from a longitudinal study
Source: J Nutr Health Aging. 2026 Jun 3;30(8):100895. doi: 10.1016/j.jnha.2026.100895 (PMC13265677; doi:10.1016/j.jnha.2026.100895)
Supplement: Supplementary file 1 [file mmc1.docx]

**Association of average cumulative sensory impairment with falls and hip fractures in older adults: evidence from a longitudinal study**

**Supplementary materials**

**Table S1** Subgroup analysis of the association between average cumulative sensory impairment and risk of falls

| Subgroups | HR, 95% CI | | |
| --- | --- | --- | --- |
|  | Dual sensory impairment | Visual sensory impairment | Auditory sensory impairment |
| Age, P for interaction | 0.237 | 0.603 | 0.166 |
| 60-64 years | 1.18 (1.09, 1.28) *** | 1.17 (1.08, 1.27) *** | 1.13 (1.04, 1.22) ** |
| ≥ 65 years | 1.11 (1.04, 1.19) ** | 1.13 (1.06, 1.22) ** | 1.06 (0.99, 1.13) |
| Sex, P for interaction | 0.861 | 0.392 | 0.338 |
| Male | 1.13 (1.05, 1.23) ** | 1.10 (1.02, 1.20) * | 1.11 (1.03, 1.20) * |
| Female | 1.15 (1.07, 1.24) *** | 1.19 (1.10, 1.28) *** | 1.07 (1.01, 1.15) * |
| Residence, P for interaction | 0.213 | 0.962 | 0.632 |
| Rural | 1.16 (1.08, 1.24) *** | 1.14 (1.07, 1.22) *** | 1.12 (1.05, 1.20) *** |
| Urban | 1.09 (1.00, 1.20) * | 1.15 (1.05, 1.27) ** | 1.02 (0.94, 1.12) |
| Marital status | 0.274 | 0.334 | 0.451 |
| Married | 1.11 (1.05, 1.18) ** | 1.12 (1.05, 1.19) *** | 1.07 (1.01, 1.13) * |
| Others | 1.26 (1.11, 1.42) *** | 1.26 (1.11, 1.42) *** | 1.16 (1.03, 1.31) * |
| Education | 0.329 | 0.957 | 0.734 |
| Illiterate | 1.11 (1.04, 1.19) ** | 1.12 (1.04, 1.20) ** | 1.07 (1.00, 1.14) |
| Junior school and below | 1.20 (1.08, 1.35) ** | 1.25 (1.12, 1.40) *** | 1.11 (1.00, 1.23) * |
| High school and above | 1.16 (1.02, 1.32) *** | 1.13 (0.99, 1.29) | 1.13 (1.00, 1.29) * |
| Smoking status | 0.444 | 0.454 | 0.597 |
| Never | 1.17 (1.09, 1.25) *** | 1.18 (1.10, 1.26) *** | 1.11 (1.03, 1.18) ** |
| Ever | 1.10 (1.01, 1.20) * | 1.11 (1.02, 1.21) * | 1.06 (0.98, 1.15) |
| Drinking status | 0.485 | 0.513 | 0.565 |
| Never | 1.17 (1.09, 1.25) *** | 1.17 (1.09, 1.26) *** | 1.11 (1.04, 1.19) ** |
| Ever | 1.10 (1.01, 1.20) * | 1.11 (1.02, 1.21) * | 1.06 (0.97, 1.15) |
| Social participation | 0.195 | 0.506 | 0.132 |
| Yes | 1.12 (1.03, 1.21) ** | 1.14 (1.06, 1.24) ** | 1.06 (0.98, 1.14) |
| No | 1.16 (1.08, 1.26) *** | 1.16 (1.07, 1.25) *** | 1.12 (1.04, 1.20) * |
| Chronic disease | 0.435 | 0.796 | 0.159 |
| 0-1 chronic condition | 1.13 (1.05, 1.21) ** | 1.16 (1.08, 1.25) ** | 1.06 (0.99, 1.14) |
| ≥ 2 chronic conditions | 1.15 (1.06, 1.24) ** | 1.12 (1.03, 1.22) ** | 1.12 (1.03, 1.21) * |

All models were adjusted for age, sex, residence, marital status, education, smoking status, drinking status, social participation, hypertension, heart disease, stroke, chronic lung disease, arthritis or rheumatism, kidney disease, digestive disease, diabetes, and memory-related disease. HR, hazard ratio. *P < 0.05; **P < 0.01; ***P < 0.001.

**Table S2** Subgroup analysis of the association between average cumulative sensory impairment and risk of hip fractures

| Subgroups | HR, 95% CI |  |  |
| --- | --- | --- | --- |
|  | Dual sensory impairment | Visual sensory impairment | Auditory sensory impairment |
| Age, P for interaction | 0.890 | 0.071 | 0.192 |
| 60-64 years | 1.39 (1.05, 1.82) *** | 1.57 (1.18, 2.10) ** | 1.15 (0.88, 1.47) |
| ≥ 65 years | 1.47 (1.19, 1.82) *** | 1.20 (0.98, 1.47) | 1.52 (1.24, 1.86) ** |
| Sex, P for interaction | 0.489 | 0.998 | 0.273 |
| Male | 1.32 (1.05, 1.68) * | 1.31 (1.03, 1.65) * | 1.22 (0.97, 1.52) |
| Female | 1.52 (1.20, 1.92) ** | 1.32 (1.05, 1.66) * | 1.47 (1.17, 1.84) ** |
| Residence, P for interaction | 0.708 | 0.293 | 0.173 |
| Rural | 1.36 (1.12, 1.66) ** | 1.37 (1.13, 1.47) ** | 1.22 (1.02, 1.47) * |
| Urban | 1.53 (1.10, 2.11) * | 1.19 (0.87, 1.56) | 1.63 (1.19, 2.25) ** |
| Marital status | 0.528 | 0.437 | 0.109 |
| Married | 1.41 (1.16, 1.71) ** | 1.40 (1.14, 1.71) ** | 1.27 (1.05, 1.52) * |
| Others | 1.52 (1.10, 2.11) * | 1.15 (0.85, 1.56) | 1.69 (1.22, 2.33) ** |
| Education | 0.499 | 0.349 | 0.795 |
| Illiterate | 1.39 (1.15, 1.69) ** | 1.27 (1.05, 1.54) * | 1.34 (1.12, 1.62) ** |
| Junior school and below | 1.23 (0.85, 1.79) | 1.28 (0.87, 1.86) | 1.12 (0.79, 1.59) |
| High school and above | 2.01 (1.09, 3.74) * | 1.85 (1.01, 3.42) * | 1.68 (0.94, 3.00) |
| Smoking status | 0.497 | 0.769 | 0.436 |
| Never | 1.49 (1.19, 1.88) ** | 1.34 (1.07, 1.69) * | 1.41 (1.14, 1.75) *** |
| Ever | 1.31 (1.02, 1.68) * | 1.27 (0.99, 1.61) | 1.62 (0.97, 2.55) |
| Drinking status | 0.156 | 0.623 | 0.078 |
| Never | 1.63 (1.28, 2.06) *** | 1.39 (1.10, 1.75) ** | 1.55 (1.24, 1.94) *** |
| Ever | 1.22 (0.96, 1.54) | 1.22 (0.96, 1.54) | 1.14 (0.90, 1.43) |
| Social participation | 0.239 | 0.689 | 0.192 |
| Yes | 1.60 (1.25, 2.05) *** | 1.37 (1.08, 1.74) * | 1.55 (1.21, 1.92) *** |
| No | 1.30 (1.03, 1.66) * | 1.28 (1.02, 1.60) * | 1.21 (0.97, 1.50) |
| Chronic disease | 0.968 | 0.344 | 0.451 |
| 0-1 chronic condition | 1.44 (1.17, 1.77) ** | 1.26 (1.03, 1.54) * | 1.42 (1.16, 174) ** |
| ≥ 2 chronic conditions | 1.38 (1.05, 1.81) * | 1.43 (1.08, 1.89) * | 1.21 (0.93, 1.56) |

All models were adjusted for age, sex, residence, marital status, education, smoking status, drinking status, social participation, hypertension, heart disease, stroke, chronic lung disease, arthritis or rheumatism, kidney disease, digestive disease, diabetes, and memory-related disease. *P < 0.05; **P < 0.01; ***P < 0.001.

**Table S3** Association between average cumulative sensory impairment and fall risks using full dataset analysis

| Sensory impairment | Falls,  Cases/Events | HR, 95% CI | | |  |
| --- | --- | --- | --- | --- | --- |
|  |  | Model 1 | Model 2 | Model 3 | Model 4 |
| Dual sensory impairment |  |  |  |  |  |
| Quartile 1 | 317/837 | Reference | Reference | Reference | Reference |
| Quartile 2 | 346/795 | 1.20 (1.03, 1.40) * | 1.18 (1.01, 1.37) * | 1.18 (1.01, 1.37) * | 1.13 (0.97, 1.31) |
| Quartile 3 | 429/862 | 1.40 (1.21, 1.62) *** | 1.38 (1.20, 1.60) *** | 1.38 (1.19, 1.54) *** | 1.29 (1.11, 1.50) ** |
| Quartile 4 | 453/827 | 1.60 (1.39, 1.85) *** | 1.51 (1.30, 1.74) *** | 1.51 (1.31, 1.75) *** | 1.36 (1.17, 1.58) *** |
| Test for linear trend | - | 1.17 (1.12, 1.22) *** | 1.15 (1.10, 1.20) *** | 1.15 (1.10, 1.20) *** | 1.11 (1.06, 1.16) *** |
| Per SD increment | - | 1.21 (1.15, 1.27) *** | 1.18 (1.12, 1.25) *** | 1.18 (1.12, 1.25) *** | 1.14 (1.08, 1.20) *** |
| Visual sensory impairment |  |  |  |  |  |
| Quartile 1 | 295/791 | Reference | Reference | Reference | Reference |
| Quartile 2 | 408/948 | 1.19 (1.02, 1.38) * | 1.20 (1.03, 1.39) * | 1.19 (1.02, 1.38) * | 1.15 (0.99, 1.33) |
| Quartile 3 | 394/779 | 1.45 (1.24, 1.68) *** | 1.43 (1.23, 1.63) *** | 1.43 (1.23, 1.66) *** | 1.32 (1.13, 1.54) *** |
| Quartile 4 | 448/803 | 1.67 (1.44, 1.93) *** | 1.56 (1.35, 1.82) *** | 1.56 (1.34, 1.81) *** | 1.43 (1.23, 1.66) *** |
| Test for linear trend | - | 1.19 (1.14, 1.24) *** | 1.16 (1.11, 1.22) *** | 1.16 (1.11, 1.22) *** | 1.13 (1.08, 1.18) *** |
| Per SD increment | - | 1.22 (1.16, 1.29) *** | 1.19 (1.13, 1.25) *** | 1.19 (1.13, 1.25) *** | 1.15 (1.09, 1.21) *** |
| Auditory sensory impairment |  |  |  |  |  |
| Quartile 1 | 320/800 | Reference | Reference | Reference | Reference |
| Quartile 2 | 373/827 | 1.19 (1.02, 1.38) * | 1.16 (1.00, 1.34) | 1.15 (0.99, 1.33) | 1.13 (0.97, 1.31) |
| Quartile 3 | 399/838 | 1.26 (1.09, 1.46) ** | 1.25 (1.08, 1.45) ** | 1.24 (1.08, 1.44) ** | 1.19 (1.02, 1.38) * |
| Quartile 4 | 453/856 | 1.43 (1.24, 1.65) *** | 1.38 (1.19, 1.59) *** | 1.37 (1.19, 1.58) *** | 1.26 (1.10, 1.47) ** |
| Test for linear trend | - | 1.12 (1.07, 1.17) *** | 1.11 (1.06, 1.16) *** | 1.11 (1.06, 1.16) *** | 1.08 (1.03, 1.13) ** |
| Per SD increment | - | 1.13 (1.08, 1.19) *** | 1.12 (1.07, 1.18) *** | 1.12 (1.07, 1.18) *** | 1.09 (1.03, 1.15) ** |

Model 1, unadjusted; Model 2, adjusted for age, sex, residence, marital status, and education; Model 3, further adjusted for smoking status, drinking status, and social participation; Model 4, additionally adjusted for hypertension, heart disease, stroke, chronic lung disease, arthritis or rheumatism, kidney disease, digestive disease, diabetes, and memory-related disease. *P < 0.05; **P < 0.01; ***P < 0.001.

**Table S**4 Association between average cumulative sensory impairment and hip fracture risks using full dataset analysis

| Sensory impairment | Hip fractures,  Cases/Events | HR, 95% CI | | |  |
| --- | --- | --- | --- | --- | --- |
|  |  | Model 1 | Model 2 | Model 3 | Model 4 |
| Dual sensory impairment |  |  |  |  |  |
| Quartile 1 | 26/837 | Reference | Reference | Reference | Reference |
| Quartile 2 | 38/795 | 1.57 (0.95, 2.59) | 1.57 (0.95, 2.59) | 1.58 (0.96, 2.60) | 1.53 (0.93, 2.53) |
| Quartile 3 | 58/862 | 2.19 (1.38, 3.48) ** | 2.13 (1.34, 3.39) ** | 2.12 (1.33, 3.36) ** | 2.07 (1.30, 3.30) ** |
| Quartile 4 | 64/827 | 2.56 (1.62, 4.04) ** | 2.23 (1.41, 3.54) ** | 2.26 (1.43, 3.57) ** | 2.15 (1.34, 3.43) ** |
| Test for linear trend | - | 1.35 (1.18, 1.54) *** | 1.28 (1.12, 1.47) *** | 1.29 (1.13, 1.47) *** | 1.27 (1.11, 1.45) ** |
| Per SD increment | - | 1.54 (1.31, 1.82) *** | 1.45 (1.23, 1.71) *** | 1.45 (1.23, 1.71) *** | 1.43 (1.21, 1.69) *** |
| Visual sensory impairment |  |  |  |  |  |
| Quartile 1 | 31/791 | Reference | Reference | Reference | Reference |
| Quartile 2 | 37/948 | 0.99 (0.61, 1.59) | 1.03 (0.66, 1.64) | 1.01 (0.63, 1.63) | 1.01 (0.60, 1.55) |
| Quartile 3 | 50/779 | 1.63 (1.04, 2.56) * | 1.61 (1.03, 2.53) * | 1.62 (1.03, 2.54) * | 1.55 (0.8, 2.44) |
| Quartile 4 | 68/803 | 2.19 (1.43, 3.35) *** | 1.91 (1.24, 2.94) ** | 1.90 (1.24, 2.93) ** | 1.83 (1.13, 2.83) ** |
| Test for linear trend | **-** | 1.35 (1.18, 1.55) ** | 1.28 (1.15, 1.46) *** | 1.28 (1.12, 1.47) *** | 1.26 (1.10, 1.44) ** |
| Per SD increment | **-** | 1.46 (1.24, 1.73) *** | 1.36 (1.15, 1.60) *** | 1.36 (1.15, 1.60) *** | 1.33 (1.13, 1.57) ** |
| Auditory sensory impairment |  |  |  |  |  |
| Quartile 1 | 31/800 | Reference | Reference | Reference | Reference |
| Quartile 2 | 41/827 | 1.30 (0.82, 2.07) | 1.27 (0.80, 2.03) | 1.28 (0.80, 2.04) | 1.25 (0.78, 2.00) |
| Quartile 3 | 50/838 | 1.55 (0.99, 2.42) | 1.59 (1.01, 2.48) * | 1.59 (1.04, 2.49) * | 1.56 (0.99, 2.45) |
| Quartile 4 | 64/856 | 1.98 (1.29, 3.04) ** | 1.78 (1.16, 2.74) ** | 1.79 (1.17, 2.76) ** | 1.72 (1.11, 2.66) * |
| Test for linear trend | - | 1.25 (1.09, 1.42) *** | 1.21 (1.06, 1.38) ** | 1.21 (1.06, 1.38) ** | 1.20 (1.05, 1.37) ** |
| Per SD increment | - | 1.40 (1.20, 1.64) *** | 1.35 (1.16, 1.58) *** | 1.36 (1.16, 1.59) *** | 1.34 (1.14, 1.57) *** |

Model 1, unadjusted; Model 2, adjusted for age, sex, residence, marital status, and education; Model 3, further adjusted for smoking status, drinking status, and social participation; Model 4, additionally adjusted for hypertension, heart disease, stroke, chronic lung disease, arthritis or rheumatism, kidney disease, digestive disease, diabetes, and memory-related disease. *P < 0.05; **P < 0.01; ***P < 0.001.

**Table S5** E-value for the association between average cumulative sensory impairment and fall risks

| Sensory impairment | E-value, 95% CI | | |  |
| --- | --- | --- | --- | --- |
|  | Model 1 | Model 2 | Model 3 | Model 4 |
| Dual sensory impairment |  |  |  |  |
| Quartile 1 | Reference | Reference | Reference | Reference |
| Quartile 2 | 1.69 (1.24, NA) | 1.64 (1.11, NA) | 1.64 (1.11, NA) | - |
| Quartile 3 | 2.15 (1.74, NA) | 2.13 (1.69, NA) | 2.10 (1.69, NA) | 1.90 (1.49, NA) |
| Quartile 4 | 2.60 (2.15, NA) | 2.41 (1.95, NA) | 2.41 (1.95, NA) | 2.06 (1.64, NA) |
| Test for linear trend | 1.62 (1.49, NA) | 1.57 (1.43, NA) | 1.59 (1.46, NA) | 1.49 (1.34, NA) |
| Per SD increment | 1.67 (1.54, NA) | 1.62 (1.46, NA) | 1.67 (1.51, NA) | 1.57 (1.40, NA) |
| Visual sensory impairment |  |  |  |  |
| Quartile 1 | Reference | Reference | Reference | Reference |
| Quartile 2 | 1.69 (1.21, NA) | 1.69 (1.24, NA) | 1.67 (1.21, NA) | - |
| Quartile 3 | 2.19 (1.74, NA) | 2.15 (1.69, NA) | 2.14 (1.69, NA) | 1.90 (1.46, NA) |
| Quartile 4 | 2.75 (2.26, NA) | 2.54 (2.06, NA) | 2.52 (2.04, NA) | 2.21 (1.76, NA) |
| Test for linear trend | 1.67 (1.54, NA) | 1.59 (1.46, NA) | 1.59 (1.46, NA) | 1.51 (1.38, NA) |
| Per SD increment | 1.76 (1.59, NA) | 1.67 (1.51, NA) | 1.67 (1.51, NA) | 1.57 (1.40, NA) |
| Auditory sensory impairment |  |  |  |  |
| Quartile 1 | Reference | Reference | Reference | Reference |
| Quartile 2 | 1.64 (1.16, NA) | - | - | - |
| Quartile 3 | 1.83 (1.40, NA) | 1.81 (1.37, NA) | 1.79 (1.37, NA) | 1.64 (1.16, NA) |
| Quartile 4 | 2.21 (1.79, NA) | 2.08 (1.67, NA) | 2.08 (1.67, NA) | 1.83 (1.40, NA) |
| Test for linear trend | 1.49 (1.34, NA) | 1.46 (1.31, NA) | 1.46 (1.31, NA) | 1.37 (1.20, NA) |
| Per SD increment | 1.51 (1.37, NA) | 1.49 (1.34, NA) | 1.49 (1.31, NA) | 1.40 (1.21, NA) |

Model 1, unadjusted; Model 2, adjusted for age, sex, residence, marital status, and education; Model 3, further adjusted for smoking status, drinking status, and social participation; Model 4, additionally adjusted for hypertension, heart disease, stroke, chronic lung disease, arthritis or rheumatism, kidney disease, digestive disease, diabetes, and memory-related disease.

**Table S6** E-value for the association between average cumulative sensory impairment and hip fracture risks

| Sensory impairment | Hip fractures,  Cases/Events | E-values, 95% CI | | |  |
| --- | --- | --- | --- | --- | --- |
|  |  | Model 1 | Model 2 | Model 3 | Model 4 |
| Dual sensory impairment |  |  |  |  |  |
| Quartile 1 | 26/846 | Reference | Reference | Reference | Reference |
| Quartile 2 | 39/808 | - | - | - | - |
| Quartile 3 | 58/886 | 3.72 (2.06, NA) | 3.60 (1.97, NA) | 3.99 (1.94, NA) | 3.46 (1.83, NA) |
| Quartile 4 | 66/851 | 4.62 (2.69, NA) | 3.93 (2.19, NA) | 4.01 (2.26, NA) | 3.74 (2.06, NA) |
| Test for linear trend | - | 2.04 (1.64, NA) | 2.04 (1.64, NA) | 1.88 (1.51, NA) | 1.86 (1.46, NA) |
| Per SD increment | - | 2.45 (1.95, NA) | 2.26 (1.76, NA) | 2.26 (1.76, NA) | 2.21 (1.71, NA) |
| Visual sensory impairment |  |  |  |  |  |
| Quartile 1 | 32/799 | Reference | Reference | Reference | Reference |
| Quartile 2 | 37/964 | - | - | - | - |
| Quartile 3 | 51/802 | 2.54 (1.16, NA) | 2.52 (1.11, NA) | 2.54 (1.11, NA) | - |
| Quartile 4 | 69/864 | 3.66 (2.13, NA) | 3.06 (1.69, NA) | 3.07 (1.69, NA) | 2.87 (1.51, NA) |
| Test for linear trend | **-** | 2.01 (1.62, NA) | 1.83 (1.46, NA) | 1.86 (1.46, NA) | 1.79 (1.40, NA) |
| Per SD increment | **-** | 2.2 (1.76, NA) | 2.04 (1.57, NA) | 2.13 (1.57, NA) | 1.97 (1.49, NA) |
| Auditory sensory impairment |  |  |  |  |  |
| Quartile 1 | 31/810 | Reference | Reference | Reference | Reference |
| Quartile 2 | 41/844 | - | - | - | - |
| Quartile 3 | 50/859 | - | - | - | - |
| Quartile 4 | 67/878 | 3.52 (2.01, NA) | 3.06 (1.67, NA) | 3.10 (1.71, NA) | 2.94 (1.57, NA) |
| Test for linear trend | - | 1.83 (1.46, NA) | 1.74 (1.34, NA) | 1.74 (1.34, NA) | 1.71 (1.31, NA) |
| Per SD increment | - | 2.19 (1.71, NA) | 2.06 (1.51, NA) | 2.08 (1.62, NA) | 2.04 (1.57, NA) |

Model 1, unadjusted; Model 2, adjusted for age, sex, residence, marital status, and education; Model 3, further adjusted for smoking status, drinking status, and social participation; Model 4, additionally adjusted for hypertension, heart disease, stroke, chronic lung disease, arthritis or rheumatism, kidney disease, digestive disease, diabetes, and memory-related disease.

Table S7 Association between average cumulative sensory impairment and hip fracture risks after further adjusted for medications use

| Sensory impairment | HR and 95% CI for falls | HR and 95% CI for hip fractures |
| --- | --- | --- |
| Dual sensory impairment |  |  |
| Quartile 1 | Reference | Reference |
| Quartile 2 | 1.12 (0.96, 1.30) | 1.56 (0.95, 2.57) |
| Quartile 3 | 1.29 (1.11, 1.49) ** | 2.02 (1.26, 3.23) ** |
| Quartile 4 | 1.35 (1.17, 1.57) *** | 2.18 (1.36, 3.47) ** |
| Test for linear trend | 1.11 (1.06, 1.16) ** | 1.27 (1.11, 1.45) ** |
| Per SD increment | 1.14 (1.08, 1.20) *** | 1.42 (1.21, 1.69) *** |
| Visual sensory impairment |  |  |
| Quartile 1 | Reference | Reference |
| Quartile 2 | 1.15 (0.99, 1.33) | 0.97 (0.60, 1.56) |
| Quartile 3 | 1.29 (1.11, 1.50) ** | 1.50 (0.96, 2.34) |
| Quartile 4 | 1.43 (1.23, 1.66) *** | 1.75 (1.13, 2.69) ** |
| Test for linear trend | 1.12 (1.07, 1.18) *** | 1.24 (1.09, 1.43) ** |
| Per SD increment | 1.14 (1.08, 1.19) *** | 1.32 (1.12, 1.55) ** |
| Auditory sensory impairment |  |  |
| Quartile 1 | Reference | Reference |
| Quartile 2 | 1.12 (0.96, 1.30) | 1.24 (0.78, 1.99) |
| Quartile 3 | 1.18 (1.02, 1.37) * | 1.53 (0.97, 2.41) |
| Quartile 4 | 1.26 (1.09, 1.45) ** | 1.78 (1.15, 2.74) * |
| Test for linear trend | 1.08 (1.03, 1.13) ** | 1.19 (1.04, 1.36) ** |
| Per SD increment | 1.09 (1.03, 1.14) ** | 1.32 (1.13, 1.55) *** |

All models were adjusted for age, sex, residence, marital status, education, smoking status, drinking status, social participation, hypertension, heart disease, stroke, chronic lung disease, arthritis or rheumatism, kidney disease, digestive disease, diabetes, memory-related disease, and medications use for hypertension, diabetes, and dyslipidemia. *P < 0.05; **P < 0.01; ***P < 0.001.

**
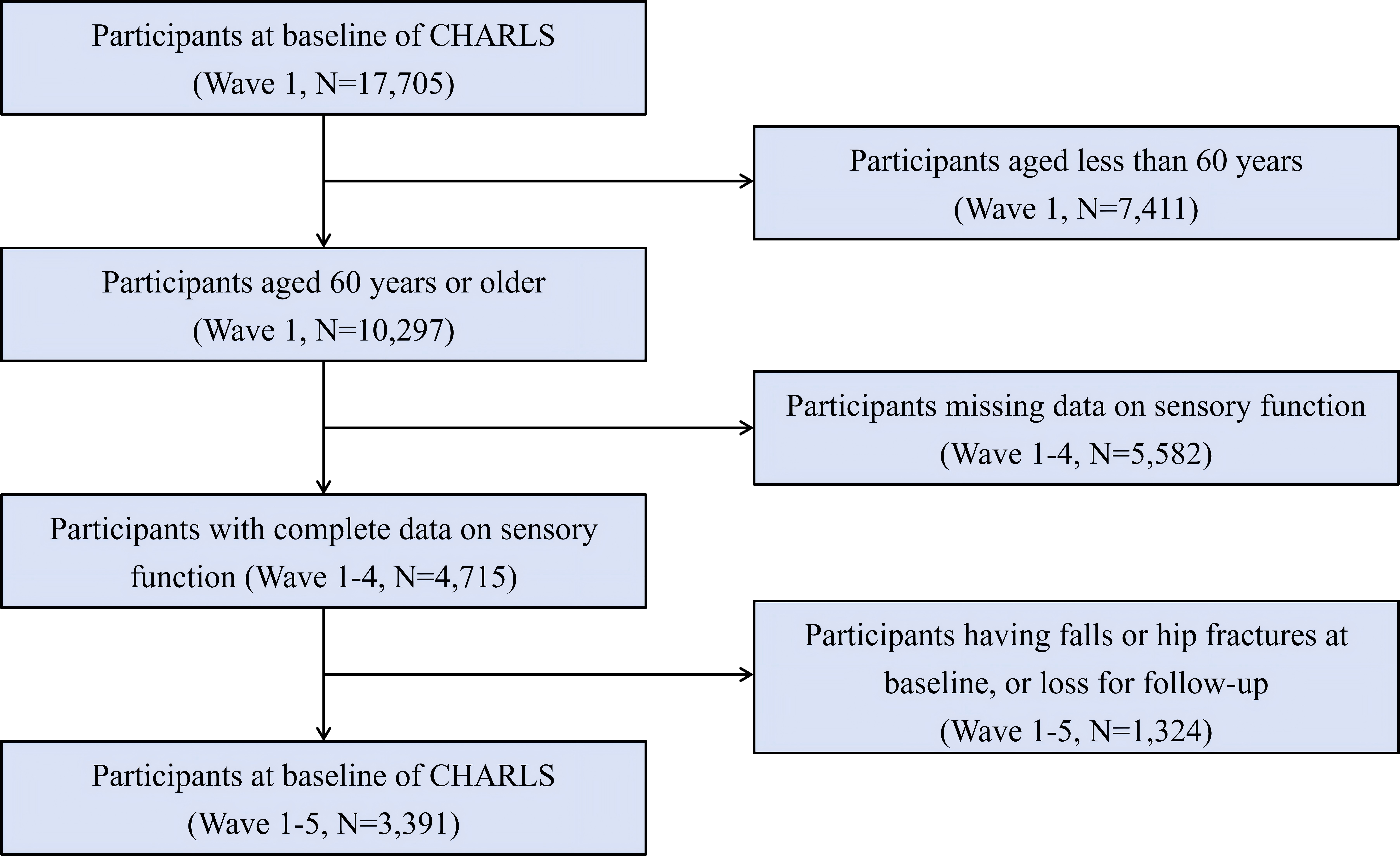
Figure S1** Flowchart of participant selection

**
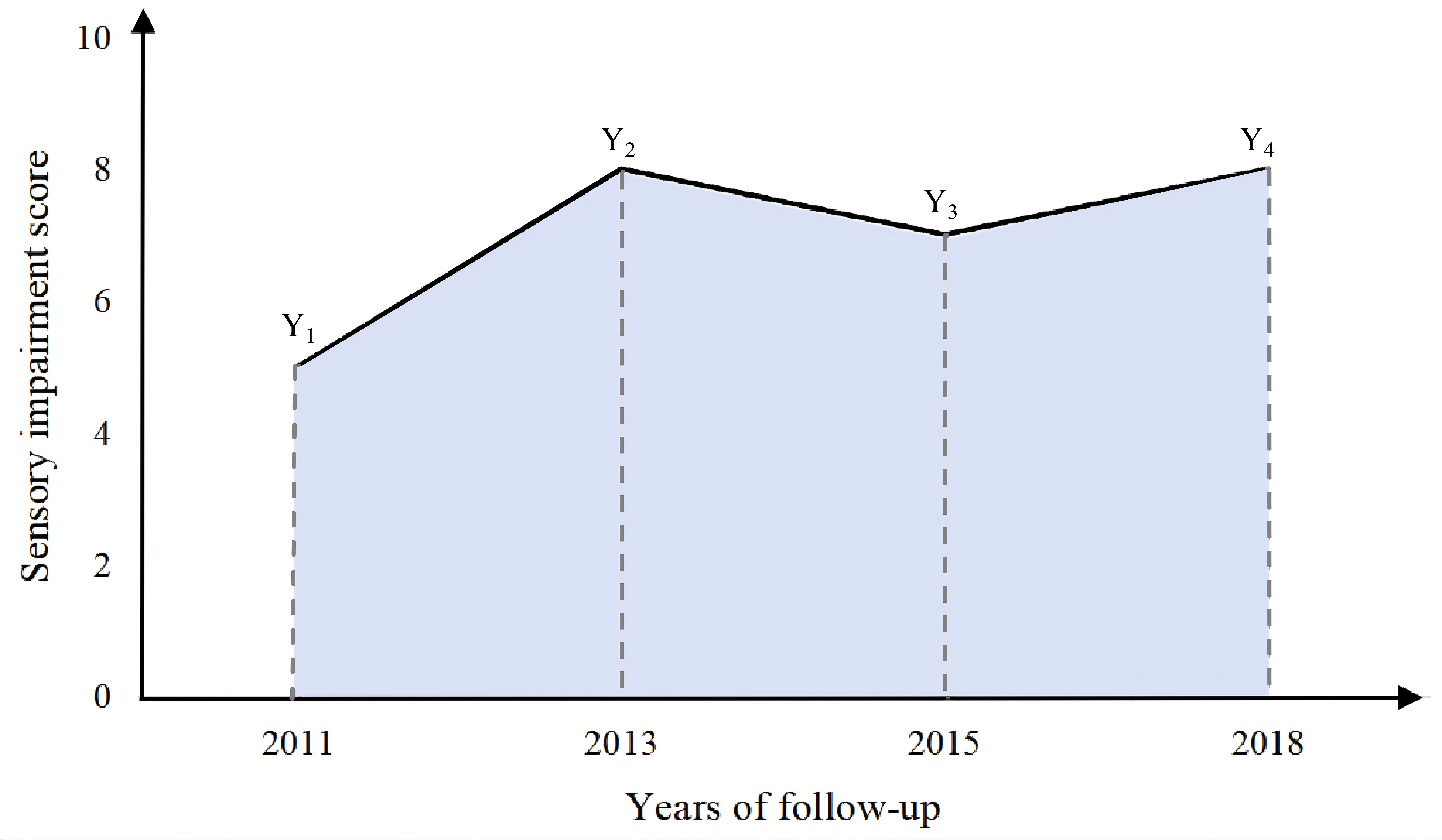
**

**Figure S2** Average cumulative sensory impairment score
